# The impact of career planning on health behaviors in medical students: a mixed methods study

**DOI:** 10.3389/fmed.2026.1815494

**Published:** 2026-05-28

**Authors:** Yuanlong Huang, Chunsong Yang, Bingyao Kang, Siyi He, Jingya Zhou, Le Yang

**Affiliations:** 1 Department of Gastroenterology,Chengdu Xinhua Hospital Affiliated to North Sichuan Medical College, Chengdu, Sichuan, China; 2 Department of Pharmacy, Evidence-based Pharmacy Center, West China Second Hospital, Sichuan University, Chengdu, China; 3 Key Laboratory of Birth Defects and Related Diseases of Women and Children, Ministry of Education, Sichuan University, Chengdu, China; 4 Department of Pediatric Clinic, West China Second University Hospital, Sichuan University, Chengdu, China; 5 School of Public Health, Gansu University of Chinese Medicine, Lanzhou, China; 6 Academic Affairs Office, West China Second Hospital, Sichuan University, Chengdu, China; 7 School of Marxism, Sichuan University, Chengdu, China

**Keywords:** career planning, health behaviors, life satisfaction, medical students, mixed methods study

## Abstract

**Aims:**

Career planning, life satisfaction, and health behaviors are key factors in the physical and psychological development of medical students. However, the complex interrelationship among these factors remains unclear. We aimed to explore the intrinsic relationship between career planning, life satisfaction, and health behaviors in Chinese medical students, and to analyze the mediating role of life satisfaction.

**Methods:**

This study employs a mixed-methods design, collecting data through a cross-sectional study. The sample consists of university students aged 18 to 24 from universities in Eastern, Central, and Western China. Semi-structured interviews further explored the interaction among these factors. Life satisfaction was measured with the SWLS scale, career planning with the CPPS scale, and health behaviors with the HPLP-II scale.

**Results:**

A total of 3,335 medical students from 34 universities across 14 provinces in China were included in the study, including 1,408 nursing students, 1,280 pharmacy students, 600 clinical medical students, 47 students in other medical disciplines. The scores for health behaviors, life satisfaction, and career planning were 61.48 ± 15.05, 22.90 ± 6.57, and 50.28 ± 12.76, respectively, all showing significant positive correlations. Medical students generally faced significant academic pressure, particularly from academic performance and employment prospects. Additionally, 77.5% of students planned for graduate school or postgraduate studies, while 52.9% considered their current major not their intended career field. Path analysis revealed that the various dimensions of career awareness and planning significantly influenced health behaviors both directly and indirectly, with life satisfaction serving as a partial mediator between career planning and health behaviors. Qualitative results validated the five dimensions of career awareness and planning, emphasizing the importance of information gathering, self-awareness, career preparation, and social support. Life satisfaction and health behaviors were found to be closely linked with career planning, with adaptability and planning clarity playing a critical role in life satisfaction and health behaviors.

**Conclusion:**

Chinese medical students often lack career planning guidance, which affects their health behaviors. Career awareness can improve health through increased life satisfaction. Collaboration among individuals, families, universities, and authorities is needed to support students’ career development and well-being.

## 1 Background

China is one of the most populous countries in the world. As an important subgroup of the youth population, medical students are large in number and shoulder a critical mission in the future development of the healthcare system. With changes in medical education models and the evolving healthcare environment, medical students face increasingly severe challenges in academic demands, career development, and mental health (1, 2). Intense coursework, frequent examinations, pressure from clinical rotations, and uncertainty regarding future employment and career prospects place medical students at high risk for mental health problems (1, 3, 4). A meta-analysis on anxiety symptoms among medical students worldwide reported an overall prevalence of 33.8%, with the prevalence in Asian medical students reaching 35.2%, significantly higher than the global average (4). Domestic studies in China have also indicated prominent mental health problems among undergraduate medical students, with reported prevalence rates of anxiety ranging from 8.54 to 88.30% (mean 27.22%) and depression ranging from 13.10 to 76.21% (mean 32.74%) (5).

At the same time, unhealthy behaviors are common among medical students, including insufficient physical activity, irregular daily schedules, and unbalanced diets (6–8). Studies have shown that approximately 40% of medical students fail to meet the recommended levels of physical activity, and nearly half report irregular eating habits or insufficient sleep (6–9). In addition, long-term academic stress and unhealthy lifestyles are closely associated with an increased risk of overweight and obesity among medical students (9), which is particularly pronounced during the clinical stage.

In response to these health issues among medical students, career planning has gradually gained attention as a psychological intervention (10). Career planning not only helps adolescents clarify their career goals but also reduces academic and social pressure, enhances self-efficacy, alleviates anxiety, and promotes mental health and the development of healthy behaviors (11–14). In recent years, the role of career planning in mental health has been widely studied (13, 14). However, there is still a lack of sufficient empirical research on how career planning influences adolescent health behaviors, especially through the mediating role of life satisfaction.

Career planning, life satisfaction, and health behaviors are critical factors in the physical and psychological development of adolescents, and there exists a complex interrelationship among these three factors. In career development theory, career planning is considered to enhance an individual’s self-efficacy, helping them better cope with future challenges, thus improving life satisfaction (15, 16). In China, adolescents face external pressures such as the college entrance exam and employment, making the role of career planning particularly important. It not only helps adolescents clarify their career goals but also alleviates anxiety caused by career uncertainty (17–19). Through career planning, adolescents can reduce psychological stress, enhance their self-efficacy, and motivate them to actively engage in healthy behaviors. According to social cognitive theory, self-efficacy is a key driver of behavior (20, 21). A clear career goal not only strengthens adolescents’ confidence in facing challenges but also enhances their commitment to and execution of health behaviors (10, 22). Career planning helps adolescents manage their time efficiently, maintain healthy eating and sleeping habits, and foster healthy behavior (19, 22).

From the perspective of positive psychology, life satisfaction (including personal happiness and self-identity) has a direct relationship with an individual’s behavior (23, 24). Adolescents with high life satisfaction are typically more likely to engage in positive health behaviors such as regular exercise, healthy eating, and good sleep (25). Therefore, life satisfaction is not only an important indicator of mental health but also a promoter of health behaviors. Career planning, by enhancing adolescents’ self-efficacy, not only alleviates academic pressure and improves life satisfaction but also effectively promotes the development of healthy behaviors.

Although existing literature has explored the relationship between career planning and mental health, or the influence of life satisfaction on health behaviors (11, 13, 19, 26), few studies have examined the comprehensive interaction between career planning, life satisfaction, and health behaviors. The primary objective of this study is to examine the relationship between career planning, life satisfaction, and health behaviors among medical students. Secondary objectives include exploring the influence of specific dimensions of career planning on life satisfaction, evaluating the mediating role of life satisfaction in the association between career planning and health behaviors, and qualitatively investigating students’ experiences and perspectives regarding these relationships, with the goal of providing new theoretical frameworks and practical strategies for health interventions targeting adolescents.

## 2 Methods

### 2.1 Research design

This study adopts a mixed-methods design with an explanatory sequential approach. Quantitative analysis is conducted first, followed by qualitative research to further explain and deepen the results. The qualitative research will use semi-structured interviews to explore the relationship between career planning, life satisfaction, and health behaviors, providing a more comprehensive understanding of the interactive effects among these factors.

### 2.2 Research participants

#### 2.2.1 Inclusion criteria

This study includes medical students aged 18–24, who are capable of understanding and voluntarily participating in the research, signing informed consent, completing questionnaires, and participating in interviews. All participants must possess basic reading and comprehension abilities. Although the study primarily targets medical students, students from related healthcare fields (nursing, pharmacy, and other medical disciplines) were included to capture the diversity of healthcare students’ career planning experiences and health behaviors, ensuring broader applicability of the findings.

#### 2.2.2 Exclusion criteria

Participants who are unwilling to participate or have not signed the consent form, those with significant psychological or physical disorders, or those who cannot participate normally for any other reasons will be excluded.

### 2.3 Sampling method

For the quantitative research, a convenience sampling method will be used. Teachers from universities in Eastern, Central, and Western China will collaborate by distributing the questionnaire and recommending appropriate students to participate in the survey to ensure sample diversity and representativeness. For the qualitative research, Participants were recruited using maximum variation sampling. Teachers from 12 universities recommended students meeting the inclusion criteria. Invitations were sent via email and WeChat, and interested students provided informed consent prior to scheduling face-to-face interviews. The study period will be from December 2024 to March 2025.

### 2.4 Data collection

#### 2.4.1 Baseline data collection

Demographic characteristics, career planning, career decision-making, and other related information will be collected through an online questionnaire.

#### 2.4.2 Measurement tools

##### 2.4.2.1 Satisfaction With Life Scale (SWLS)

SWLS is used to assess an individual’s overall life satisfaction (27), comprising 5 items with a 7-point Likert scale (1 = Strongly Disagree, 7 = Strongly Agree). Higher scores indicate greater life satisfaction.

##### 2.4.2.2 Career Perception and Planning Scale (CPPS)

The CPPS is a self-designed scale used to measure college students’ career planning cognition and practices. The scale consists of five dimensions with a total of 22 items: ① Career Perception and Information Gathering (CPIG); ② Self-awareness and Career Planning (SCP); ③ Career Preparation and Development (CPD); ④ Interpersonal Communication and Support (ICS); ⑤ Adaptation and Self-development (ASD). A 5-point Likert scale is used, with higher scores indicating better planning ability.

##### 2.4.2.3 Health-Promoting Lifestyle Profile II (HPLP-II)

HPLP-II is used to assess individual health behaviors (28), including six dimensions: Interpersonal Relations, Nutrition, Health Responsibility, Physical Activity, Stress Management, and Spiritual Growth, totaling 28 items. A 4-point Likert scale is used, with higher scores indicating more active health behaviors.

#### 2.4.3 Qualitative data collection

Data will be collected through face-to-face interviews with open-ended questions, inviting participants to share their experiences with career planning, life satisfaction, and health behaviors. Each interview will last approximately 20 to 40 min, and the interview content will be recorded and transcribed. The interviews will address core issues of career planning, perceptions of life satisfaction, and specific cases of how these factors influence health behaviors.

### 2.5 Path model design

A structural equation model (SEM) will be used for path analysis to explore the relationships between career planning, life satisfaction, and health behaviors. The specific hypotheses are: (1) The stronger the career planning, the higher the life satisfaction. (2) Life satisfaction mediates the relationship between career planning and health behaviors. (3) Life satisfaction has a positive effect on health behaviors.

### 2.6 Data analysis

#### 2.6.1 Quantitative data analysis

Data analysis will be conducted using SPSS 26.0 and AMOS 24.0 software. The steps are as follows: (1) Descriptive statistics: Analyze baseline data of the sample. (2) Reliability and validity analysis: Cronbach’s *α* coefficient will be used to evaluate internal consistency, and factor analysis will be performed to assess validity. (3) Path analysis: Structural equation modeling will be employed to validate the path hypotheses and evaluate the direct and indirect effects of career planning and life satisfaction on health behaviors.

#### 2.6.2 Qualitative data analysis

This study will use thematic analysis and NVivo software to code and categorize interview data. Through verbatim transcription and initial coding, key themes will be identified to extract the core factors influencing career planning, life satisfaction, and health behaviors. Comparative analysis will be conducted to determine how qualitative data supports quantitative findings. Thematic analysis was conducted following Braun and Clarke’s six-phase framework. Interview transcripts were read multiple times to familiarize with the data, initial codes were generated for meaningful units, codes were grouped into potential themes, themes were reviewed and refined, and final themes were defined and named. These themes were then integrated with quantitative findings to produce a comprehensive interpretation of career planning, life satisfaction, and health behaviors.

#### 2.6.3 Integration of quantitative and qualitative results

The integration of quantitative and qualitative results will be carried out through a triangulation strategy to ensure the reliability of the research conclusions. Common themes from qualitative research will be matched and compared with quantitative data, and meta-inferences will be made. Data consistency will be evaluated in three ways: confirmation (data results support each other), extension (differences provide a deeper understanding), and inconsistency (which indicates contradictions that need further exploration).

### 2.7 Ethical considerations

This study follows the ethical principles outlined in the Declaration of Helsinki and has been approved by the Ethics Committee of West China Second University Hospital, Sichuan University. All participants will sign informed consent forms and be informed about the voluntary nature and confidentiality of their participation in the research. During the interviews, all data will be anonymized, and psychological support resources will be provided.

## 3 Results

### 3.1 Quantitative results

#### 3.1.1 Characteristics of research participants and influencing factors (Figure 1; Table 1)

**Figure 1:**
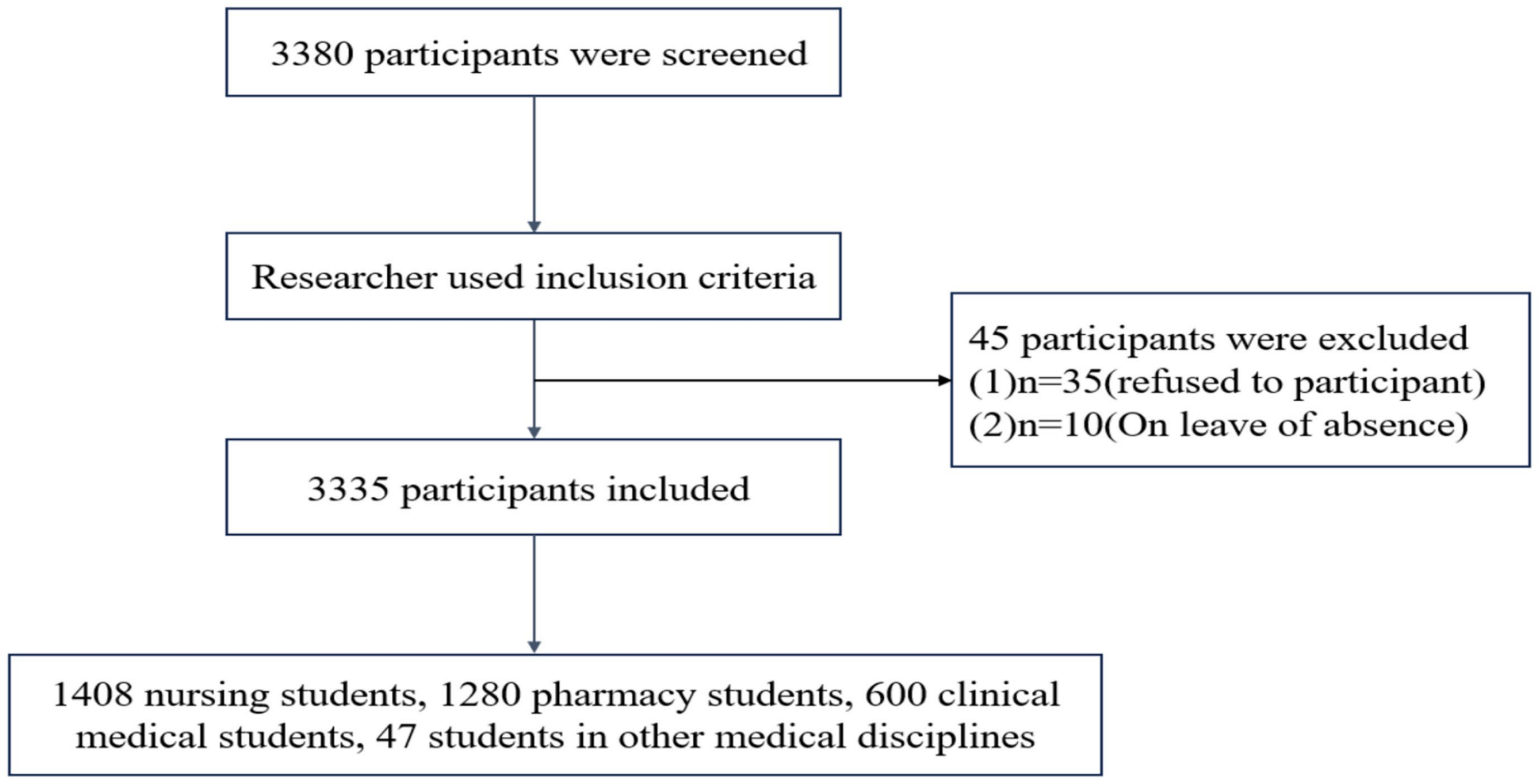
Study selection flowchart.

**Table 1:** Characteristics of the target population.

Items	*n*	HPLP
Gender		
Male	1,947 (58.4%)	61.42 ± 14.86
Female	1,388 (41.6%)	61.56 ± 15.33
Age*	18.56 ± 0.979	
Region*		
East	494 (14.8%)	59.89 ± 14.69
Central	807 (24.2%)	61.03 ± 15.26
West	2,034 (61.0%)	62.04 ± 15.03
Grade*		
Freshman (Grade 1)	2,884 (86.5%)	62.93 ± 15.28
Sophomore (Grade 2)	294 (8.8%)	54.05 ± 10.10
Junior (Grade 3)	66 (2.0%)	48.26 ± 4.63
Senior (Grade 4)	77 (2.3%)	48.66 ± 5.79
Grade 5	14 (0.4%)	50.00 ± 6.40
Course pressure*		
High	1,653 (49.6%)	62.01 ± 15.41
Moderate	1,478 (44.3%)	61.18 ± 14.83
Low	204 (6.1%)	59.31 ± 13.46
Other pressures besides course study		
High	902 (27.0%)	61.46 ± 14.86
Moderate	1,552 (46.5%)	62.00 ± 15.45
Low	881 (26.4%)	60.58 ± 14.51
In college life, which aspect causes you the most stress?*		
Financial Situation	262 (7.9%)	60.72 ± 15.67
Academic Performance	2,000 (60.0%)	62.89 ± 15.00
Employment Direction	818 (24.5%)	58.62 ± 14.92
Family Life	24 (0.7%)	60.58 ± 17.12
Campus Interpersonal Relationships	152 (4.6%)	61.54 ± 13.14
Other	79 (2.4%)	57.85 ± 14.07
Overall future planning*		
Apply for Exemption from Entrance Examination	1,834 (55.0%)	63.11 ± 15.15
Take the Postgraduate Entrance Examination	750 (22.5%)	60.40 ± 15.27
Study Abroad	155 (4.6%)	65.59 ± 15.71
Employment	348 (10.4%)	58.01 ± 13.94
Entrepreneurship	40 (1.2%)	58.43 ± 12.84
Uncertain	208 (6.2%)	54.33 ± 10.95
Is your current major your intended major? *		
No	1,765 (52.9%)	60.84 ± 14.26
Yes	1,570 (47.1%)	62.19 ± 15.87
When you filled out your college entrance examination (Gaokao) application, it was: *		
Completed independently by yourself	666 (20.0%)	60.00 ± 15.06
Completed independently by your parents	33 (1.0%)	62.91 ± 14.07
Completed in consultation with your parents	1,691 (50.7%)	61.68 ± 14.94
Completed jointly by you, your parents, and your teachers	690 (20.7%)	61.68 ± 15.10
Completed by a college application consulting agency	255 (7.6%)	63.26 ± 15.57
Have you ever done a professional career plan?*		
Yes	700 (21.0%)	62.78 ± 16.42
No	2,635 (79.0%)	61.13 ± 14.65
What form of career planning would you like?		
On-campus lectures	426 (12.8%)	60.99 ± 15.18
Group counseling	226 (6.8%)	61.87 ± 16.23
One-on-one consultation	1,467 (44.0%)	61.06 ± 14.62
Career experience	1,133 (34.0%)	62.23 ± 15.34
Other	83 (2.5%)	60.07 ± 14.56

*Indicates *p* < 0.05.

A total of 3,380 medical students were recruited for the study, with 45 declining to participate, resulting in 3,335 included cases (1,408 nursing students, 1,280 pharmacy students, 600 clinical medical students, 47 students in other medical disciplines) (Figure 1). Participants were from 14 provinces in Eastern, Central, and Western China, representing 34 universities. The average age of the participants was 18.56 ± 0.979 years.

(1) *Health behaviors, life satisfaction, and career planning scores*: The scores for HPLP, SWLS, and CPPS were 61.48 ± 15.05, 22.90 ± 6.57, and 50.28 ± 12.76, respectively.
(2) *Stress levels*: Survey results showed that 49.6% of students reported significant academic pressure, with the main stressors being academic performance (60.0%) and career prospects (24.5%).
(3) *Career planning and educational intentions*: 77.5% of students planned to continue their studies or pursue graduate studies, while 52.9% felt their current major was not aligned with their career aspirations. 79.0% of students had not received professional career planning guidance.
(4) *Factors influencing future career choices*: The main factors influencing future career choices were salary (86.2%), personal interest (72.5%), and career development prospects (69.5%). The univariate and multivariate analyses of health behaviors indicated that age, grade, regional differences, sources of stress, future career plans, whether the major matched the career interest, and whether career planning guidance had been received significantly affected health behaviors (Table 2).

**Table 2:** Multiple linear regression analysis of factors influencing HPLP.

Variable	Non-standardized coefficient	Standard error	Standard coefficient	*t*	*p*
Constant	69.731	2.019		34.539	0.000
Gender	−1.561	0.360	−0.102	−4.340	0.000
Region	1.189	0.339	0.058	3.507	0.000
Grade	−3.438	0.559	−0.144	−6.146	0.000
Course pressure	−0.597	0.420	−0.024	−1.421	0.156
In college life, which aspect causes you the most stress?	−0.600	0.258	−0.039	−2.323	0.020
Overall future planning	−1.109	0.176	−0.107	−6.295	0.000
Is your current major your intended major?	1.568	0.507	0.052	3.092	0.002
Gaokao application filing method:	0.037	0.221	0.003	0.169	0.866
Have you ever done a professional career plan?	−1.704	0.616	−0.046	−2.768	0.006

#### 3.1.2 Reliability and validity analysis

(1) Structural validity

All scales showed good consistency, with Cronbach’s *α* values ranging from 0.768 to 0.899 (Supplementary Table 1). The cumulative variance contribution rate of the 12 common factors was 64.604%, with all factor loadings greater than 0.4, demonstrating strong structural validity (Supplementary Tables 2–4).

(2) Confirmatory factor analysis (CFA)

Confirmatory factor analysis (CFA) indicated good model fit, with factor loadings ranging from 0.675 to 0.827. The composite reliability (CR) of all constructs was greater than 0.7, and the average variance extracted (AVE) was greater than 0.5, confirming convergent validity.

(3) Discriminant validity

Discriminant validity was also supported, as the square root of each AVE was greater than the correlations between constructs (Supplementary Tables 5–8).

#### 3.1.3 Correlation analysis

Health behavior was significantly positively correlated with life satisfaction (*r* = 0.461), career perception and information gathering (*r* = 0.474), self-awareness and career planning (*r* = 0.398), career preparation and development (*r* = 0.476), interpersonal communication and support (*r* = 0.522), and adaptation and self-development (*r* = 0.544) (*p* < 0.01). Detailed data are shown in Supplementary Table 9.

#### 3.1.4 Path and mediation effect analysis

Structural equation modeling results indicated that the five dimensions of career perception and planning significantly affected health behaviors through direct and indirect pathways (Supplementary Tables 9, 10; Table 3). Specifically, career perception and information gathering (standardized path coefficient = 0.093, *p* < 0.05), self-awareness and career planning (standardized path coefficient = 0.115, *p* < 0.05), career preparation and development (standardized path coefficient = 0.113, *p* < 0.05), interpersonal communication and support (standardized path coefficient = 0.211, *p* < 0.05), and adaptation and self-development (standardized path coefficient = 0.185, *p* < 0.05) positively impacted life satisfaction, which in turn positively affected health behaviors (standardized path coefficient = 0.156, *p* < 0.05). Mediation effect analysis showed that life satisfaction partially mediated the relationship between these dimensions and health behaviors, with significant indirect effects (Table 4).

**Table 3:** Path analysis results.

Path analysis	Estimate	S. E.	C. R.	*p*	STD estimate
SWLS<---CPIG	0.166	0.047	3.53	***	0.093
SWLS<---SCP	0.21	0.036	5.878	***	0.115
SWLS<---CPD	0.201	0.047	4.299	***	0.113
SWLS<---ICS	0.351	0.044	8.028	***	0.211
SWLS<---ASD	0.322	0.055	5.887	***	0.185
HPLP<---CPIG	0.123	0.016	7.605	***	0.183
HPLP<---SCP	0.056	0.012	4.648	***	0.082
HPLP<---CPD	0.117	0.016	7.257	***	0.175
HPLP<---ICS	0.085	0.015	5.692	***	0.135
HPLP<---ASD	0.17	0.019	8.832	***	0.259
HPLP<---SWLS	0.059	0.007	8.189	***	0.156

***p < 0.05.

**Table 4:** Mediation effect test.

Effect types		Estimate	Lower	Upper	*p*
Direct effect	HPLP<---CPIG	0.123	0.086	0.156	0.014
	HPLP<---SCP	0.056	0.022	0.08	0.018
	HPLP<---CPD	0.117	0.083	0.148	0.013
	HPLP<---ICS	0.351	0.277	0.439	0.007
	HPLP<---ASD	0.17	0.13	0.216	0.007
Indirect effect	HPLP<---SWLS<---CPIG	0.01	0.004	0.015	0.011
	HPLP<---SWLS<---SCP	0.012	0.009	0.019	0.003
	HPLP<---SWLS<---CPD	0.012	0.008	0.018	0.002
	HPLP<---SWLS<---ICS	0.005	0.003	0.007	0.008
	HPLP<---SWLS<---ASD	0.019	0.012	0.026	0.01
Total effect	HPLP<---CPIG	0.133	0.096	0.167	0.012
	HPLP<---SCP	0.069	0.043	0.096	0.011
	HPLP<---CPD	0.129	0.095	0.165	0.01
	HPLP<---ICS	0.356	0.282	0.449	0.006
	HPLP<---ASD	0.189	0.146	0.233	0.008

### 3.2 Qualitative results

#### 3.2.1 Participants in the qualitative study

A total of 29 university students participated in the interviews, representing 12 different universities. Their ages ranged from 18 to 24, with 12 males and 17 females. The participants came from various disciplines: 10 in nursing students, 8 in pharmacy students, 6 clinical medical students, 5 in other medical disciplines. There were 12 freshmen, 10 sophomores, 5 juniors, and 2 seniors.

#### 3.2.2 Interpretation of qualitative results

The five dimensions of career perception and planning were validated in the interviews, supporting the structure of the scale. Respondents emphasized the importance of information gathering for clarity in career planning, validating the corresponding measurements in the scale. Interests and values drove planning, with personal preferences and social factors influencing the direction of planning, confirming the role of self-awareness. Career preparation behavior helped implement plans, and social support was seen as a key factor in executing plans, validating the scale’s measurements of interpersonal communication and career support. Respondents stressed the importance of dynamic adjustment in planning, believing that a flexible mechanism enhances adaptability, validating the necessity of adaptation and self-development in the scale. The synergy among these dimensions strengthened the overall effectiveness of planning (Table 5).

**Table 5:** Results of qualitative thematic analysis.

Dimension	Theme	Sub-theme	Interview typical case
SWLS	My life is largely in accordance with my ideals.	Alignment of Plans with Ideals	I-1:“Having a clear plan for each step prevents me from drifting aimlessly.” I-11:“Planning keeps my life organized and maintains direction through dynamic adjustments.”
	My life situation is very satisfactory.	Sense of Fulfillment and Meaning in Life	I-12:“After achieving the small goal of postgraduate entrance examination, my satisfaction with my life status has significantly improved. I-24:“When I know what I want, looking back on my life feels fulfilling and meaningful.”
	I am content with my life.	Subjective Evaluation of Life	I-7:“Planning brings a sense of direction, making my study and life more orderly and increasing my satisfaction.” I-9:“With a clear goal of joining a medical-litigation law firm, I do not follow the crowd and am satisfied with my focused academic status.”
	I can obtain what is important in life.	Sense of Achievement in Reaching Goals	I-1: “The goal of taking the civil service exam motivates me to keep exercising and studying, resulting in good physical fitness and academic foundation.” I-12: “Through planning, I accumulate clinical experience and gradually get closer to my career goal as a doctor.”
	I am unwilling to change my current life situation.	Acceptance of Current Life Path	I-6:“With family support for free choices, although the plan is vague, I am more accepting of the current life situation.” I-11:“The master’s and doctoral planning allows me to focus on cultivating research and teaching abilities, and my current life stage does not need to change.”
CPPS	CPIG	Proactive Acquisition of Career Information	I-1:“I hope the school offers guidance courses and provides alumni resources to obtain information related to civil service exams.” I-17:“I screen career directions through the process of elimination, relying on information comparison.”
	SCP	Planning Oriented by Interests and Values	I-1:“I set my goals by combining personal interests with the traditional civil service exam concept in Shandong.” I-9: “I enjoy legal debate, so I aim to join a healthcare-focused law firm.”
	CPD	Practical Actions for Accumulating Competitiveness	I-11: “I balance study and research to accumulate academic abilities for my master’s and doctoral goals.” I-12: “I maintain high GPA each semester and participate in research projects to prepare for postgraduate entrance examination and employment in top-tier hospitals.”
	ICS	Supportive Role of Social Networks	I-2: “I exchange career planning anxiety with friends to obtain experience and advice.” I-12: “I consult senior students for study methods to avoid detours in career preparation.”
	ASD	Dynamic Adjustment Capability of Planning	I-1: “I adjust the details of my plan according to changes in values to maintain the applicability of the goals.” I-17: “I adjust the priority of further education and employment according to changes in the job market.”
HPLP	Interpersonal Relations	The Health Benefits of Social Support	I-2: “Communicating with friends relieves anxiety and enhances coping abilities.” I-12: “Family and friends are a strong support in coping with stress, providing emotional support and solutions.”
	Nutrition	Proactive Management of a Healthy Diet	I-1: “Using delicious food to regulate emotions, balancing nutritional and psychological needs.” I-12: “Increased intake of vitamin B-rich foods like whole wheat bread and bananas to regulate the nervous system.”
	Health Responsibility	Autonomous Awareness of Healthy Behaviors	I-11: “Incorporated health management into career planning to lay the foundation for long-term goals.” I-12: “Gave up staying up late to be competent for the job of a doctor, maintaining 7–8 h of high-quality sleep.”
	Physical Activity	The Stress-Relief and Health Functions of Exercise	I-1: “Running and fitness to relieve stress and improve physical fitness.” I-26: “Running and sweating to relieve stress and regulate physical and mental state.”
	Stress Management	The Stress-Relief and Health Functions of Exercise	I-1: “Making decisive choices to reduce anxiety and avoid overthinking.” I-12: “Breaking down tasks to enhance a sense of achievement and reduce perceived stress.”
	Spiritual Growth	Pursuit of Mental Well-being	I-6: “Seeking upward through psychology books to enhance psychological resilience.” I-9: “Planning reduces anxiety and gains inner peace.”

The thematic analysis of life satisfaction confirmed the findings from the interviews, showing a close relationship between career planning and life satisfaction, exhibiting dynamic features. Clear planning helped reduce aimlessness in life, aligning daily choices with long-term goals, enhancing the sense of fulfillment and meaning in life, thus validating the scale’s effectiveness. Clearer planning also improved subjective life satisfaction and promoted resource accumulation, furthering the achievement of important life goals. Additionally, individuals with clear plans were more likely to accept their current life trajectory, while those with unclear plans might rely on external support to maintain acceptance, supplementing the scale’s measurements. The interviews also revealed the need for dynamic adaptation, emphasizing the role of flexible adjustment in reinforcing life satisfaction, which provided an additional dimension to the scale.

The six dimensions of health-promoting lifestyle were validated in the interviews, aligning well with the scale’s results. Respondents viewed social support as central to stress coping, emphasized self-management of diet and health responsibility, validating the corresponding dimensions of the scale. Exercise was seen as improving both physical and emotional health, creating a positive feedback loop, stress management strategies increased efficiency, and spiritual growth solidified overall health. The interviews also highlighted the inherent connection between health-promoting lifestyle and career planning and life satisfaction, showing that the clarity of planning influences the systematic nature of health behaviors, further supporting the relationship among the scales.

#### 3.2.3 Integration of quantitative and qualitative results

The interviews confirmed the validity of the scale structure from the quantitative study, with content from the interviews deeply corroborating the scale dimensions. The five core items of the life satisfaction scale were highly consistent with the statements from the interviewees, and dynamic adaptability was identified as a potential dimension supplement. The five dimensions of the career perception and planning scale were verified through information gathering, self-awareness, career preparation, interpersonal support, and dynamic adjustment strategies, further highlighting the synergistic mechanism of the planning system. The six dimensions of the health-promoting lifestyle scale precisely matched the health management logic of the interviewees, confirming the validity of health behavior measurements.

The interviews also revealed the complex interrelationships among variables, clarifying the positive impact of career planning on life satisfaction, and the mediating role of life satisfaction between career planning and health behaviors. Planning clarity, goal achievement, and dynamic adaptability enhanced satisfaction, and high satisfaction facilitated proactive health management. The five dimensions of career planning indirectly influenced health behaviors through information, self-drive, and interpersonal support, forming a chain of “planning → satisfaction → health behaviors,” with perceived stress being identified as a potential mediating variable. The interviews also uncovered some differences from the scale dimensions. The synergistic effects in career planning suggest that the strength of individual dimensions can affect the overall outcome, and the dynamic adaptability dimension in life satisfaction suggests that planning flexibility may modulate the strength of the path. Differences in health behaviors indicated that family support, types of stress sources, and other factors might cause heterogeneity in the relationships among variables.

## 4 Discussion

### 4.1 Key findings of this study

This study, using a combination of quantitative and qualitative methods, is the first to systematically integrate career planning, life satisfaction, and health behaviors into the same theoretical framework for analysis. The results confirm the complex relationships among these factors. The study shows that academic pressure and the ambiguity of career planning are two major challenges faced by university students. Approximately 49.6% of medical students experience significant academic pressure, with uncertainty about academic performance and unclear employment prospects being the primary sources of stress. This phenomenon reflects the dual dilemma students face between academic burdens and future career choices. Furthermore, 52.9% of medical students are dissatisfied with their current major and feel confused about their future development direction. This uncertainty may increase anxiety, thus affecting life satisfaction. Around 79% of students reported not receiving professional career planning guidance, which leads to a lack of systematic support and professional advice when making career decisions. These students often find themselves in a state of confusion and anxiety about their career paths, and emotional distress further influences their health behaviors.

Structural equation modeling indicates that various dimensions of career cognition and planning significantly affect life satisfaction, which in turn impacts health behaviors. The adequacy of information acquisition reduces career uncertainty and enhances life satisfaction. Self-awareness helps students understand their strengths and interests, thus improving life satisfaction. Interpersonal support provides emotional and resource support for career planning, promoting healthy behaviors. Adaptation and self-development highlight the flexibility of career planning, helping students maintain a positive attitude and improve health behaviors. Qualitative interviews confirm the findings from the structural equation model, with respondents generally agreeing that clear career planning reduces uncertainty, enhances life satisfaction, and improves health behaviors. Career cognition and self-awareness are seen as key factors, while interpersonal support provides necessary resources, and career planning flexibility and adaptability help maintain long-term satisfaction and health behaviors. The qualitative analysis supplements the quantitative results, providing deeper insights into the role of career planning in driving life satisfaction and health behaviors, with life satisfaction playing a mediating role between career cognition/planning and health behaviors.

From a biopsychosocial perspective, clear career planning can positively influence health behaviors through multiple mechanisms. Biologically, reduced uncertainty and academic stress lower physiological stress responses, supporting better sleep, nutrition, and physical activity. Psychologically, career clarity enhances self-efficacy, self-awareness, and a sense of purpose, motivating proactive engagement in health-promoting behaviors. Socially, career planning often involves seeking guidance and support from peers, mentors, and family, which provides both emotional and practical resources to maintain healthy habits. Furthermore, the relationship may be bidirectional: engaging in positive health behaviors can improve energy, cognitive function, and emotional resilience, which in turn supports more effective career planning. This reciprocal process suggests a reinforcing cycle where career planning and health behaviors mutually promote one another, contributing to greater life satisfaction and overall well-being.

### 4.2 Comparison with other studies

Compared with existing studies, this research provides a more comprehensive perspective on the relationships among career cognition, life satisfaction, and health behaviors. Many early studies have explored the impact of career cognition on life satisfaction, with research indicating that clarity in career goals can improve individual life satisfaction (15, 16, 29). However, fewer studies have focused on how career cognition indirectly influences health behaviors through life satisfaction. Through structural equation modeling, this study finds that life satisfaction plays an important mediating role between career cognition and health behaviors. This finding fills a gap in the research, particularly in understanding the mechanisms affecting health behaviors. Moreover, most studies focus on quantitative analysis, neglecting qualitative exploration (11, 19, 29). In contrast, this study enriches the quantitative data through qualitative interviews, revealing the practical operations of career cognition and self-awareness in career planning and their impact on students’ psychological state. Previous research has highlighted the importance of career planning for students’ mental health (11–14, 17, 19), but this study further shows that career cognition not only influences planning outcomes but also promotes positive changes in health behaviors by improving life satisfaction. This multi-level analysis helps educators better understand the mechanisms of career cognition. Unlike other studies, this study also considers the dynamic nature of career planning, emphasizing the importance of individual adaptability and self-development. Career planning is not a static goal-setting process but should be continuously adjusted based on individual development and changes in the external environment. This perspective provides a valuable supplement to existing career planning theories.

### 4.3 Practical implications

This study reveals that university students face dual challenges of academic pressure and unclear career planning, which has significant implications for individuals, families, universities, and educational authorities. (1) *At the individual and family level*: Medical students should actively engage in self-awareness and career exploration by using career assessments to clarify their interests and abilities, and by participating in internships to gain experience (19). They should also learn time management and emotional regulation to reduce academic pressure, focus on mental health (24), and enhance life satisfaction and health behaviors. Families should offer psychological support and understanding, maintain open communication, encourage career choices based on interests, and assist in career development by sharing experiences and providing internship opportunities. (2) *At the university level*: Universities should optimize course offerings, balance academic pressure with career planning education, incorporate career planning into mandatory courses, and provide personalized career guidance. Universities could establish career counseling centers, invite industry experts to give lectures, and help students clarify their career goals. Additionally, they should strengthen mental health education by offering emotional management courses and psychological counseling to help students cope with stress (30). Collaborating with companies to provide internship opportunities and vocational skills training would enhance students’ employability, alleviate job-related pressure, and improve life satisfaction. (3) *At the educational authority level*: Educational authorities should develop policies to support universities in providing mental health and career planning education, increase funding, and establish evaluation mechanisms. They should encourage collaboration between universities, share educational resources, and organize cross-university training activities. By integrating social resources and partnering with psychological counseling institutions and industry associations, educational authorities can provide students with psychological support and internship opportunities, help improve their career cognition and planning abilities, and enhance their life satisfaction and health behaviors. Through coordinated efforts between individuals, families, universities, and educational authorities, academic pressure and career confusion among students can be effectively alleviated, improving mental health and enhancing career development, thereby laying a foundation for future career paths.

### 4.4 Study limitations and future directions

The limitations of this study are as follows: (1) *Sampling method*: This study used a convenience sampling method, which may limit the representativeness and generalizability of the sample. However, this study included students from different regions of China, providing a good reflection of the situation of Chinese university students. Future studies should use random sampling methods to improve the external validity of the results. (2) Cultural and social context: Due to cultural and social differences, the findings of this study may not directly apply to university students in other countries or regions. Future research can expand this study to different cultural contexts and explore how different social systems affect career planning, life satisfaction, and health behaviors among students. (3) *Self-report data*: This study mainly relies on self-report data, which may be subject to social desirability bias, where respondents adjust their answers based on social norms or expectations. Future research could combine multiple data collection methods to improve the objectivity and accuracy of the data.

## 5 Conclusion

Chinese medical students face significant academic pressure and employment challenges, while career planning guidance is relatively insufficient. Their health behaviors are influenced by various factors. Career cognition and planning not only directly impact health behaviors but also indirectly promote improvements in health behaviors by enhancing life satisfaction. Therefore, it is recommended that personal, family, university, and educational authorities collaborate to assist students in engaging in self-awareness, clarifying career goals, and providing necessary psychological health support, thereby effectively improving students’ health behaviors.

## Data Availability

The raw data supporting the conclusions of this article will be made available by the authors, without undue reservation.
