# Supplementary material for: The impact of career planning on health behaviors in medical students: a mixed methods study
Source: Front Med (Lausanne). 2026 May 28;13:1815494. doi: 10.3389/fmed.2026.1815494 (PMC13253392; doi:10.3389/fmed.2026.1815494)
Supplement: Supplementary file 1 [file Data_Sheet_1.PDF]

Supplementary Table 1 Cronbach Reliability Analysis

	Item	Corrected Item-Total Correlation (CITC)	Cronbach's Alpha if Item Deleted	Cronbach's Alpha
SWLS	SWLS1	0.749	0.877	0.899
	SWLS2	0.749	0.877	
	SWLS3	0.741	0.879	
	SWLS4	0.746	0.878	
	SWLS5	0.761	0.874	
CPIG CPPS1-3	CPPS1	0.603	0.686	0.768
	CPPS2	0.609	0.679	
	CPPS3	0.591	0.7	
SCP CPPS4-10	CPPS4	0.751	0.879	0.899
	CPPS5	0.696	0.886	
	CPPS6	0.687	0.887	
	CPPS7	0.682	0.887	
	CPPS8	0.745	0.88	
	CPPS9	0.692	0.886	
	CPPS10	0.681	0.887	
CPD CPPS11-13	CPPS11	0.6	0.689	0.768
	CPPS12	0.597	0.692	
	CPPS13	0.605	0.683	
ICS CPPS14-19	CPPS14	0.706	0.859	0.882
	CPPS15	0.686	0.862	
	CPPS16	0.691	0.861	
	CPPS17	0.687	0.862	
	CPPS18	0.691	0.861	
	CPPS19	0.684	0.862	
ASD CPPS20-22	CPPS20	0.624	0.696	0.78
	CPPS21	0.617	0.703	
	CPPS22	0.611	0.709	
Interpersonal Relations，HPLP1-5	HPLP1	0.677	0.79	0.835
	HPLP2	0.618	0.807	
	HPLP3	0.633	0.803	
	HPLP4	0.614	0.808	
	HPLP5	0.637	0.802	
Nutrition，HPLP6-9	HPLP6	0.637	0.763	0.813
	HPLP7	0.636	0.763	
	HPLP8	0.629	0.766	
	HPLP9	0.623	0.769	
Health Responsibility，HPLP10-14	HPLP10	0.695	0.821	0.857
	HPLP11	0.658	0.831	
	HPLP12	0.659	0.831	
	HPLP13	0.65	0.833	
	HPLP14	0.697	0.821	
Physical Activity，HPLP15-18	HPLP15	0.639	0.77	0.817
	HPLP16	0.643	0.768	
	HPLP17	0.641	0.769	
	HPLP18	0.628	0.775	
Stress Management，HPLP19-23	HPLP19	0.699	0.819	0.856
	HPLP20	0.661	0.829	
	HPLP21	0.625	0.838	
	HPLP22	0.646	0.832	
	HPLP23	0.72	0.813	
Spiritual Growth，HPLP24-28	HPLP24	0.669	0.799	0.839
	HPLP25	0.625	0.811	
	HPLP26	0.617	0.813	
	HPLP27	0.621	0.812	

HPLP28	0.678	0.796

Note: SWLS: Satisfaction With Life Scale; CPPS: Career Perception and Planning Scale; HPLP: Health-Promoting Lifestyle Profile; CPIG, Career Perception and Information Gathering(CPPS1-3); SCP, Self-awareness and Career Planning (CPPS4-10); CPD, Career Preparation and Development (CPPS11-13); ICS, Interpersonal Communication and Support(CPPS14-19); ASD, Adaptation and Self-development(CPPS20-22); Interpersonal Relations (HPLP1-5); Nutrition (HPLP6-9); Health Responsibility(HPLP10-14); Physical Activity(HPLP15-18); Stress Management (HPLP19-23); Spiritual Growth (HPLP24-28).

CPPS 1: I keep up-to-date with the latest developments in my career field by reading professional books and subscribing to electronic news.

CPPS 2: I use social media and career websites to obtain various information about career development.

CPPS 3: I attend online career lectures or webinars to broaden my understanding of the industry.

CPPS 4: Through discussions with classmates, I gain a better understanding of my strengths and unique qualities.

CPPS 5: I am willing to communicate with others and listen to their opinions to understand my personality strengths and weaknesses and make corresponding adjustments.

CPPS 6: I am able to identify my personal interests and strengths from past experiences and make a rational assessment of my abilities.

CPPS 7: I have set clear short-term and long-term goals for my career future and have planned a path to achieve them.

CPPS 8: I document my career goals and plans and regularly review and revise them.

CPPS 9: I have set career goals to achieve within three years after graduation and am working towards them.

CPPS 10: I have set long-term goals for my career and take practical steps to achieve them gradually.

CPPS 11: I can create a resume showcasing my strengths and skills to help me stand out in job applications.

CPPS 12: I enhance my professional knowledge and competitiveness by participating in professional courses and self-directed learning.

CPPS 13: I actively seek and enroll in training programs that can improve my abilities and strengthen my competitiveness for future career development.

CPPS 14: I regularly interact and share experiences with classmates who can support my career development to expand my network.

CPPS 15: I continuously improve my interpersonal skills through participation in social activities and communication skills training.

CPPS 16: I actively build connections with people who can provide support and assistance for my career development.

CPPS 17: I maintain contact with teachers who can provide valuable advice for my career, learning from their experience.

CPPS 18: I communicate with relatives who have industry resources to obtain industry information and advice.

CPPS 19: Through recommendations from friends and colleagues, I indirectly meet professionals who are helpful for my career development.

CPPS 20: Over the past year, I have gained new insights into my career direction and abilities and have made self-adjustments accordingly.

CPPS 21: Recently, I have developed a different understanding of changes in the job market and career trends and have adjusted my expectations.

CPPS 22: Over the past year, I have reassessed my career goals based on personal experiences and environmental changes and made corresponding adjustments.

Supplementary Table 2 KMO and Bartlett's Test

KMO		0.960
Bartlett's Test of Sphericity	Approximate Chi-Square	87820.392
	df	1485
	p value	0

Supplementary Table 3 Table of Variance Explained

Factor Number	Eigenvalue			Variance Explained Before Rotation			Variance Explained After Rotation		
	Eigenvalue	Variance Explained (%)	Cumulative (%)	Eigenvalue	Variance Explained (%)	Cumulative (%)	Eigenvalue	Variance Explained (%)	Cumulative (%)
1	15.168	27.579	27.579	15.168	27.579	27.579	4.627	8.413	8.413
2	3.378	6.141	33.72	3.378	6.141	33.72	3.989	7.252	15.665
3	2.867	5.213	38.933	2.867	5.213	38.933	3.629	6.599	22.264
4	2.521	4.583	43.516	2.521	4.583	43.516	3.331	6.057	28.321
5	2.169	3.943	47.459	2.169	3.943	47.459	3.22	5.855	34.175

6	1.801	3.274	50.733	1.801	3.274	50.733	3.208	5.833	40.008
7	1.545	2.809	53.542	1.545	2.809	53.542	3.098	5.632	45.64
8	1.433	2.606	56.149	1.433	2.606	56.149	2.484	4.516	50.156
9	1.304	2.371	58.52	1.304	2.371	58.52	2.224	4.044	54.2
10	1.236	2.247	60.767	1.236	2.247	60.767	1.973	3.587	57.787
11	1.103	2.006	62.773	1.103	2.006	62.773	1.969	3.579	61.367
12	1.008	1.833	64.606	1.008	1.833	64.606	1.781	3.238	64.604
13	0.669	1.216	65.821	-	-	-	-	-	-
14	0.634	1.152	66.973	-	-	-	-	-	-
15	0.606	1.102	68.075	-	-	-	-	-	-
16	0.583	1.06	69.135	-	-	-	-	-	-
17	0.574	1.043	70.178	-	-	-	-	-	-
18	0.554	1.007	71.185	-	-	-	-	-	-
19	0.54	0.982	72.166	-	-	-	-	-	-
20	0.527	0.958	73.124	-	-	-	-	-	-
21	0.521	0.948	74.072	-	-	-	-	-	-
22	0.515	0.936	75.008	-	-	-	-	-	-
23	0.509	0.926	75.934	-	-	-	-	-	-
24	0.504	0.917	76.851	-	-	-	-	-	-
25	0.502	0.912	77.763	-	-	-	-	-	-
26	0.497	0.903	78.666	-	-	-	-	-	-
27	0.492	0.894	79.56	-	-	-	-	-	-
28	0.487	0.885	80.445	-	-	-	-	-	-
29	0.483	0.878	81.323	-	-	-	-	-	-
30	0.478	0.869	82.191	-	-	-	-	-	-
31	0.473	0.861	83.052	-	-	-	-	-	-
32	0.463	0.841	83.893	-	-	-	-	-	-
33	0.462	0.84	84.733	-	-	-	-	-	-
34	0.456	0.829	85.562	-	-	-	-	-	-
35	0.452	0.822	86.384	-	-	-	-	-	-
36	0.448	0.814	87.198	-	-	-	-	-	-
37	0.439	0.797	87.995	-	-	-	-	-	-
38	0.435	0.791	88.786	-	-	-	-	-	-
39	0.431	0.784	89.57	-	-	-	-	-	-
40	0.425	0.772	90.342	-	-	-	-	-	-
41	0.42	0.764	91.106	-	-	-	-	-	-
42	0.412	0.749	91.855	-	-	-	-	-	-
43	0.406	0.738	92.593	-	-	-	-	-	-
44	0.403	0.734	93.327	-	-	-	-	-	-
45	0.397	0.721	94.048	-	-	-	-	-	-
46	0.389	0.708	94.756	-	-	-	-	-	-
47	0.38	0.69	95.446	-	-	-	-	-	-
48	0.362	0.658	96.104	-	-	-	-	-	-
49	0.356	0.647	96.751	-	-	-	-	-	-
50	0.34	0.619	97.369	-	-	-	-	-	-
51	0.338	0.614	97.983	-	-	-	-	-	-
52	0.328	0.596	98.579	-	-	-	-	-	-
53	0.317	0.576	99.155	-	-	-	-	-	-
54	0.291	0.528	99.684	-	-	-	-	-	-
55	0.174	0.316	100	-	-	-	-	-	-

Supplementary Table 4 Table of rotated Factor Loadings

Item	Factor Loadings Coefficient												Common variance
	Factor 1	Factor 2	Factor 3	Factor 4	Factor 5	Factor 6	Factor 7	Factor 8	Factor 9	Factor 10	Factor 11	Factor 12	
SWLS1	0.123	0.16	**0.784**	0.08	0.058	0.062	0.111	0.082	0.101	0.059	0.068	0.083	0.713

SWLS2	0.14	0.152	**0.777**	0.096	0.098	0.067	0.087	0.101	0.057	0.057	0.11	0.084	0.713
SWLS3	0.114	0.143	**0.791**	0.067	0.077	0.065	0.114	0.062	0.075	0.077	0.05	0.057	0.708
SWLS4	0.131	0.135	**0.781**	0.088	0.093	0.068	0.096	0.102	0.071	0.086	0.083	0.06	0.709
SWLS5	0.13	0.155	**0.791**	0.084	0.089	0.074	0.088	0.078	0.083	0.079	0.064	0.092	0.726
CPPS1	0.121	0.178	0.108	0.078	0.065	0.099	0.105	0.069	0.109	**0.751**	0.109	0.098	0.691
CPPS2	0.121	0.18	0.121	0.076	0.085	0.117	0.119	0.104	0.086	**0.739**	0.105	0.1	0.688
CPPS3	0.135	0.174	0.108	0.084	0.07	0.119	0.1	0.085	0.087	**0.733**	0.089	0.112	0.668
CPPS4	**0.808**	0.071	0.071	0.058	0.071	0.069	0.085	0.053	0.026	0.042	0.073	0.059	0.698
CPPS5	**0.748**	0.11	0.105	0.018	0.079	0.064	0.055	0.041	0.055	0.062	0.072	0.049	0.612
CPPS6	**0.737**	0.114	0.093	0.049	0.086	0.046	0.083	0.051	0.08	0.045	0.048	0.042	0.599
CPPS7	**0.736**	0.105	0.089	0.031	0.078	0.079	0.049	0.064	0.084	0.076	0.004	0.065	0.597
CPPS8	**0.801**	0.076	0.063	0.03	0.073	0.073	0.088	0.063	0.026	0.054	0.087	0.063	0.691
CPPS9	**0.739**	0.107	0.095	0.052	0.1	0.067	0.048	0.066	0.046	0.065	0.067	0.062	0.605
CPPS10	**0.73**	0.113	0.101	0.041	0.095	0.068	0.055	0.049	0.033	0.049	0.054	0.081	0.59
CPPS11	0.135	0.158	0.106	0.081	0.082	0.102	0.11	0.082	0.079	0.103	**0.748**	0.114	0.686
CPPS12	0.139	0.183	0.108	0.068	0.126	0.116	0.102	0.091	0.105	0.097	**0.724**	0.089	0.67
CPPS13	0.114	0.179	0.138	0.061	0.091	0.102	0.095	0.092	0.075	0.104	**0.744**	0.111	0.687
CPPS14	0.13	**0.736**	0.141	0.083	0.077	0.106	0.126	0.094	0.063	0.107	0.076	0.064	0.652
CPPS15	0.146	**0.72**	0.117	0.071	0.074	0.092	0.091	0.071	0.089	0.089	0.077	0.134	0.626
CPPS16	0.116	**0.714**	0.131	0.094	0.105	0.067	0.106	0.106	0.063	0.115	0.099	0.11	0.627
CPPS17	0.136	**0.711**	0.135	0.073	0.088	0.09	0.105	0.105	0.073	0.111	0.113	0.084	0.623
CPPS18	0.117	**0.724**	0.148	0.082	0.072	0.097	0.124	0.088	0.077	0.071	0.08	0.103	0.632
CPPS19	0.111	**0.72**	0.116	0.084	0.088	0.075	0.089	0.065	0.105	0.09	0.125	0.106	0.623
CPPS20	0.163	0.231	0.154	0.102	0.101	0.116	0.108	0.107	0.14	0.121	0.133	**0.699**	0.701
CPPS21	0.156	0.234	0.141	0.118	0.086	0.095	0.116	0.097	0.112	0.124	0.125	**0.707**	0.695
CPPS22	0.177	0.219	0.133	0.103	0.129	0.124	0.131	0.101	0.093	0.132	0.123	**0.689**	0.684
HPLP1	0.094	0.1	0.076	0.055	0.106	**0.763**	0.122	0.084	0.116	0.035	0.069	**0.06**	0.665
HPLP2	0.069	0.107	0.065	0.089	0.132	**0.693**	0.123	0.134	0.074	0.092	0.059	**0.071**	0.581
HPLP3	0.113	0.086	0.058	0.1	0.122	**0.717**	0.109	0.093	0.115	0.05	0.051	**0.058**	0.604
HPLP4	0.086	0.076	0.043	0.076	0.115	**0.686**	0.151	0.108	0.121	0.096	0.046	**0.076**	0.572
HPLP5	0.08	0.096	0.074	0.07	0.102	**0.715**	0.127	0.104	0.137	0.068	0.1	0.021	0.608
HPLP6	0.104	0.111	0.11	0.088	0.142	0.129	0.146	**0.717**	0.15	0.06	0.078	0.084	0.655
HPLP7	0.112	0.134	0.122	0.096	0.148	0.147	0.187	**0.698**	0.087	0.115	0.084	0.054	0.651
HPLP8	0.083	0.151	0.098	0.102	0.14	0.147	0.168	**0.698**	0.145	0.058	0.052	0.065	0.638
HPLP9	0.094	0.117	0.111	0.099	0.134	0.16	0.165	**0.685**	0.158	0.058	0.082	0.08	0.625
HPLP10	0.113	0.133	0.095	0.101	0.148	0.169	**0.726**	0.124	0.13	0.09	0.082	0.051	0.677
HPLP11	0.101	0.138	0.131	0.111	0.14	0.156	**0.672**	0.156	0.145	0.074	0.043	0.102	0.617
HPLP12	0.102	0.139	0.125	0.109	0.172	0.135	**0.664**	0.139	0.167	0.08	0.083	0.079	0.613
HPLP13	0.099	0.151	0.111	0.111	0.153	0.136	**0.666**	0.159	0.142	0.049	0.111	0.06	0.607
HPLP14	0.103	0.125	0.108	0.12	0.167	0.169	**0.721**	0.141	0.104	0.103	0.059	0.074	0.678
HPLP15	0.097	0.143	0.099	0.13	0.168	0.188	0.204	0.152	**0.663**	0.09	0.094	0.088	0.649
HPLP16	0.108	0.13	0.117	0.124	0.153	0.184	0.203	0.184	**0.653**	0.112	0.091	0.098	0.646
HPLP17	0.105	0.146	0.113	0.113	0.143	0.196	0.171	0.137	**0.685**	0.1	0.092	0.095	0.662
HPLP18	0.105	0.116	0.15	0.153	0.18	0.197	0.188	0.182	**0.632**	0.072	0.063	0.095	0.627
HPLP19	0.048	0.062	0.068	**0.8**	0.019	0.08	0.081	0.071	0.041	0.047	0.077	0.026	0.68
HPLP20	0.054	0.07	0.069	**0.764**	0.032	0.058	0.082	0.032	0.079	0.041	0.035	0.079	0.625
HPLP21	0.047	0.096	0.056	**0.727**	0.039	0.063	0.093	0.084	0.08	0.053	0.018	0.04	0.576
HPLP22	0.03	0.093	0.08	**0.747**	0.043	0.078	0.073	0.061	0.069	0.058	0.003	0.067	0.604
HPLP23	0.054	0.069	0.078	**0.813**	0.008	0.072	0.079	0.068	0.081	0.019	0.063	0.03	0.702
HPLP24	0.083	0.094	0.075	0.012	**0.762**	0.106	0.135	0.094	0.067	0.026	0.044	0.093	0.656
HPLP25	0.119	0.093	0.071	0.046	**0.707**	0.129	0.125	0.094	0.098	0.052	0.057	0.014	0.586
HPLP26	0.115	0.084	0.085	0.037	**0.682**	0.13	0.151	0.122	0.09	0.063	0.069	0.049	0.568
HPLP27	0.106	0.064	0.071	0.038	**0.716**	0.099	0.104	0.082	0.112	0.044	0.084	0.05	0.585
HPLP28	0.13	0.102	0.082	0.018	**0.758**	0.101	0.111	0.105	0.102	0.043	0.04	0.059	0.66

Rotation Method: Varimax (Maximum Variance Method)

Supplementary Table 5 Table of Model Fit Indices

Indicators	CMIN/DF	RMR	GFI	NFI	TLI	CFI	RMSEA
Optimal Values	<3	<0.05	>0.9	>0.9	>0.9	>0.9	<0.08
Acceptable Values	<5	<0.08	>0.8	>0.8	>0.8	>0.8	<0.1
Measured Results	2.327	0.017	0.964	0.964	0.977	0.979	0.02

Supplementary Table 6: Factor Loading Table

Items			Estimate	S.E.	C.R.	P	STD Estimate
CPPS 1	<---	CPIG	1.013	0.03	34.195	***	0.723
CPPS 2	<---	CPIG	1.058	0.031	34.662	***	0.74
CPPS 3	<---	CPIG	1				0.711
CPPS 4	<---	SCP	1.147	0.026	44.907	***	0.827
CPPS 5	<---	SCP	1.023	0.026	39.669	***	0.727
CPPS 6	<---	SCP	1.015	0.026	39.006	***	0.715
CPPS 7	<---	SCP	1.01	0.026	38.777	***	0.71
CPPS 8	<---	SCP	1.156	0.026	44.733	***	0.824
CPPS 9	<---	SCP	1.026	0.026	39.417	***	0.722
CPPS 10	<---	SCP	1				0.71
CPPS 11	<---	CPD	0.997	0.029	34.514	***	0.718
CPPS 12	<---	CPD	1.008	0.029	34.747	***	0.726
CPPS 13	<---	CPD	1				0.728
CPPS 14	<---	ICS	1.04	0.025	42.399	***	0.759
CPPS 15	<---	ICS	1.004	0.024	41.259	***	0.739
CPPS 16	<---	ICS	1.014	0.024	41.685	***	0.746
CPPS 17	<---	ICS	1.014	0.024	41.413	***	0.742
CPPS 18	<---	ICS	1.002	0.024	41.563	***	0.744
CPPS 19	<---	ICS	1				0.735
CPPS 20	<---	ASD	1.018	0.027	37.794	***	0.748
CPPS 21	<---	ASD	0.992	0.027	37.061	***	0.729
CPPS 22	<---	ASD	1				0.731
SWLS5	<---	SWLS	1				0.814
SWLS4	<---	SWLS	0.994	0.019	51.143	***	0.797
SWLS3	<---	SWLS	0.967	0.019	50.404	***	0.788
SWLS2	<---	SWLS	1.005	0.019	51.626	***	0.802
SWLS1	<---	SWLS	0.985	0.019	51.407	***	0.8
HPLP1	<---	Interpersonal Relations	1				0.751
HPLP2	<---	Interpersonal Relations	0.916	0.024	37.485	***	0.691
HPLP3	<---	Interpersonal Relations	0.927	0.024	38.267	***	0.705
HPLP4	<---	Interpersonal Relations	0.91	0.024	37.333	***	0.688
HPLP5	<---	Interpersonal Relations	0.934	0.024	38.816	***	0.715
HPLP6	<---	Nutrition	1				0.723
HPLP7	<---	Nutrition	1.022	0.027	37.713	***	0.73
HPLP8	<---	Nutrition	0.989	0.027	37.191	***	0.719
HPLP9	<---	Nutrition	0.978	0.026	37.056	***	0.716
HPLP10	<---	Health Responsibility	1				0.763
HPLP11	<---	Health Responsibility	0.953	0.023	41.259	***	0.724
HPLP12	<---	Health Responsibility	0.945	0.023	41.401	***	0.726
HPLP13	<---	Health Responsibility	0.948	0.023	40.67	***	0.714
HPLP14	<---	Health Responsibility	1.017	0.023	43.927	***	0.767
HPLP15	<---	Physical Activity	1				0.727
HPLP16	<---	Physical Activity	1.007	0.026	39.08	***	0.735
HPLP17	<---	Physical Activity	0.987	0.026	38.393	***	0.721
HPLP18	<---	Physical Activity	1	0.026	38.465	***	0.723
HPLP19	<---	Stress Management	1				0.79
HPLP20	<---	Stress Management	0.899	0.022	41.059	***	0.711
HPLP21	<---	Stress Management	0.833	0.022	38.746	***	0.675

HPLP22	<---	Stress Management	0.864	0.022	40.096	***	0.696
HPLP23	<---	Stress Management	1.036	0.022	47.201	***	0.81
HPLP24	<---	Spiritual Growth	1				0.75
HPLP25	<---	Spiritual Growth	0.92	0.025	37.54	***	0.69
HPLP26	<---	Spiritual Growth	0.919	0.025	37.355	***	0.687
HPLP27	<---	Spiritual Growth	0.915	0.025	37.147	***	0.683
HPLP28	<---	Spiritual Growth	1.026	0.025	41.365	***	0.763

Supplementary Table 7 Convergent Validity

**Dimension**	CR	AVE
SWLS	0.899	0.64
CPIG	0.768	0.525
SCP	0.899	0.562
CPD	0.768	0.524
ICS	0.882	0.554
ASD	0.78	0.542
Interpersonal Relations	0.836	0.505
Nutrition	0.813	0.521
Health Responsibility	0.858	0.546
Physical Activity	0.817	0.528
Stress Management	0.856	0.545
Spiritual Growth	0.84	0.512

Supplementary Table 8 Discriminant Validity

	CPD	SWLS	ICS	SCP	CPIG	ASD	Interpersonal Relations	Nutrition	Health Responsibility	Physical Activity	Spiritual Growth	Stress Management
CPD	0.724											
SWLS	0.433***	0.8										
ICS	0.549***	0.491***	0.744									
SCP	0.392***	0.364***	0.399***	0.749								
CPIG	0.519***	0.422***	0.555***	0.380***	0.725							
ASD	0.596***	0.498***	0.650***	0.458***	0.593***	0.736						
Interpersonal Relations	0.429***	0.322***	0.406***	0.320***	0.435***	0.458***	0.71					
Nutrition	0.469***	0.434***	0.490***	0.348***	0.464***	0.522***	0.542***	0.722				
Health Responsibility	0.480***	0.444***	0.511***	0.363***	0.488***	0.536***	0.564***	0.647***	0.739			
Physical Activity	0.512***	0.461***	0.516***	0.367***	0.527***	0.599***	0.632***	0.683***	0.701***	0.727		
Spiritual Growth	0.397***	0.343***	0.383***	0.356***	0.348***	0.430***	0.459***	0.514***	0.546***	0.555***	0.716	
Stress Management	0.282***	0.288***	0.312***	0.189***	0.294***	0.357***	0.304***	0.348***	0.376***	0.414***	0.177***	0.738

Supplementary Table 9 Correlation Analysis

Items	SWLS	CPIG	SCP	CPD	ICS	ASD	HPLP
SWLS	1						
CPIG	0.350**	1					
SCP	0.335**	0.322**	1				
CPD	0.359**	0.398**	0.329**	1			
ICS	0.436**	0.457**	0.364**	0.452**	1		
ASD	0.416**	0.459**	0.390**	0.461**	0.539**	1	
HPLP	0.461**	0.474**	0.398**	0.476**	0.522**	0.544**	1

* $p<0.05$ ** $p<0.01$

Supplementary Table 10 Structural Equation Model Fit Index Analysis

Indicator	CMIN/DF	RMR	GFI	NFI	TLI	CFI	RMSEA
Optimal Indicator	<3	<0.05	>0.9	>0.9	>0.9	>0.9	<0.08
Acceptable Indicator	<5	<0.08	>0.8	>0.8	>0.8	>0.8	<0.1
Measurement Result	2.384	0.022	0.962	0.962	0.976	0.978	0.02
